# Authors’ reply

**Published:** 2010-10

**Authors:** P. Sreejith, V. Jha, H. S. Kohli, M. Rathi, K. L. Gupta, V. Sakhuja

**Affiliations:** Department of Nephrology, Postgraduate Institute of Medical Education and Research, Chandigarh-160 012, India

Sir,

We appreciate the keen interest[1] in our case report “Allograft and prostatic involvement in a renal transplant recipient with disseminated tuberculosis: a case report”[2] and very relevant issues raised. In this regard, we would like to bring to your attention that:

1. The KDIGO clinical practice guidelines for the care of kidney transplant recipients[3] recommend susceptibility testing on all isolates of *Mycobacterium tuberculosis* from kidney transplant recipients because the recipients may belong to diverse geographic locations where the prevalence of drug resistance may vary. Identifying drug susceptibility is all the more important in transplant recipients in view of the limited drug options available for them due to significant interaction of rifamycins with calcinurin inhibitors (CNIs) and mTOR inhibitors. The current recommendation for treatment of tuberculosis in kidney transplant recipient is to use the same treatment regimen as used in local general population who require therapy.[3] Rifampicin may be substituted with rifabutin with close monitoring of CNI drug levels, or a fluoroquinolone may be used instead of rifamycins. The efficacy of rifamycin sparing regimen in kidney transplant recipients has been reported from our center in the past.[4,5] The duration of therapy has not been defined, but it may be prudent to consider the experience from rifampicin-free regimen used in general population, which in general are 9 months or more in duration.
2. Aguado *et al*.[6] has reported that treatment duration less than 9 months was associated with greater mortality, and Park *et al*.[7] reported that the only factor that was significantly associated with greater recurrence of TB was duration of treatment: no recurrence was observed in patients who received more than 12 months treatment, irrespective of whether the treatment regimen included rifampicin. The Spanish Society of Infectious Disease and Clinical Microbiology recommends[8] duration of at least 12-18 months in solid organ transplant recipients.
3. The prostatic abscess in our patient was asymptomatic and was picked up only on imaging; he had no evidence of bladder outlet obstruction. Hence, an intervention for draining the same was not considered necessary. A repeat CT scan after 3 months of antitubercular chemotherapy showed complete resolution of the abscess.
